# Bilateral tension pneumothoraxes in buffalo chest several months after Nuss procedure for pectus excavatum

**DOI:** 10.1186/s13019-022-02055-7

**Published:** 2022-12-16

**Authors:** Hyung Joo Park, Chan Beom Park, Jin Yong Jeong

**Affiliations:** 1 Department of Thoracic and Cardiovascular Surgery, Seoul St. Mary’s Hospital, College of Medicine, The Catholic University of Korea, Seoul, Republic of Korea; 2 Department of Thoracic and Cardiovascular Surgery, Incheon St. Mary’s Hospital, College of Medicine, The Catholic University of Korea, Seoul, Republic of Korea; 3 Department of Thoracic and Cardiovascular Surgery, Incheon St. Mary’s Hospital, College of Medicine, The Catholic University of Korea, 56 Dongsu-ro, Bupyeong-gu, Incheon, 21431 Republic of Korea

**Keywords:** Primary spontaneous pneumothorax, Tension pneumothorax, Pectus excavatum, Nuss procedure

## Abstract

Primary spontaneous pneumothorax usually occurs in tall and thin young people without an underlying disease or traumatic history. Most patients with pectus excavatum have similar body shapes as patients with pneumothorax. Haller indices of the patients with pneumothorax and pectus excavatum are higher than normal. Pectus excavatum may be a predisposing factor for the development of primary pneumothorax. The Nuss procedure involves inserting a metal bar through the substernal space to correct the pectus excavatum, resulting in a buffalo chest in which both pleural cavities communicate with each other. Therefore, if pneumothorax occurs after the Nuss procedure, it can occur bilaterally. Recently, we encountered a life-threatening case of bilateral tension pneumothoraxes after the Nuss procedure for pectus excavatum, which were not related to surgical complications.

Dear Sir,

We read with great interest the report by Huang et al. presenting the association between primary spontaneous pneumothorax (PSP) and pectus excavatum (PE) [1]. They reviewed computed tomography (CT) images of 471 PE patients and found bleb formation in 123 patients (26.5%) and pneumothorax incidence rate of 5.6% in the bleb group and 0.5% in the non-bleb group. Another report showed a 7.83-fold increased risk of PSP incidence in the PE group compared to the non-PE group [2]. PSP can be life-threatening when it occurs as bilateral pneumothoraxes or tension pneumothorax. Recently, we encountered a life-threatening case of bilateral tension pneumothoraxes after the Nuss procedure for PE, which was not related to surgical complications.

A 13-year-old male presented with aggravating dyspnea for 5 days and a blurred vision two hours before evaluation. He had undergone a Nuss procedure for PE with a Haller index of 3.12 four and a half months ago. The surgery had been performed by inserting four metal bars due to the broad chest depression and rebound pectus carinatum. He recovered normally without surgical complications including pneumothorax (Fig. 1A) and was followed up with outpatient care until visiting the emergency room. A chest radiograph during a visit to the emergency room showed a severe collapse of the lungs on both sides compressing the heart (Fig. 1B). Chest tubes were immediately inserted into both thoracic cavities. A chest CT scan after tube insertion showed no bleb, and the left pneumothorax improved, but the right pneumothorax persisted. An operation was performed first on the right side using a thoracoscopy and revealed several bullae in the upper lobe of the right lung, and a partial pulmonary resection was performed (Fig. 1C, D). After the right lung resection, the operation was completed because there was no air leakage through both chest tubes. He was discharged with good recovery progress three days later and visited our hospital for a year without surgical complications and recurrence of pneumothorax.

**Fig. 1:**
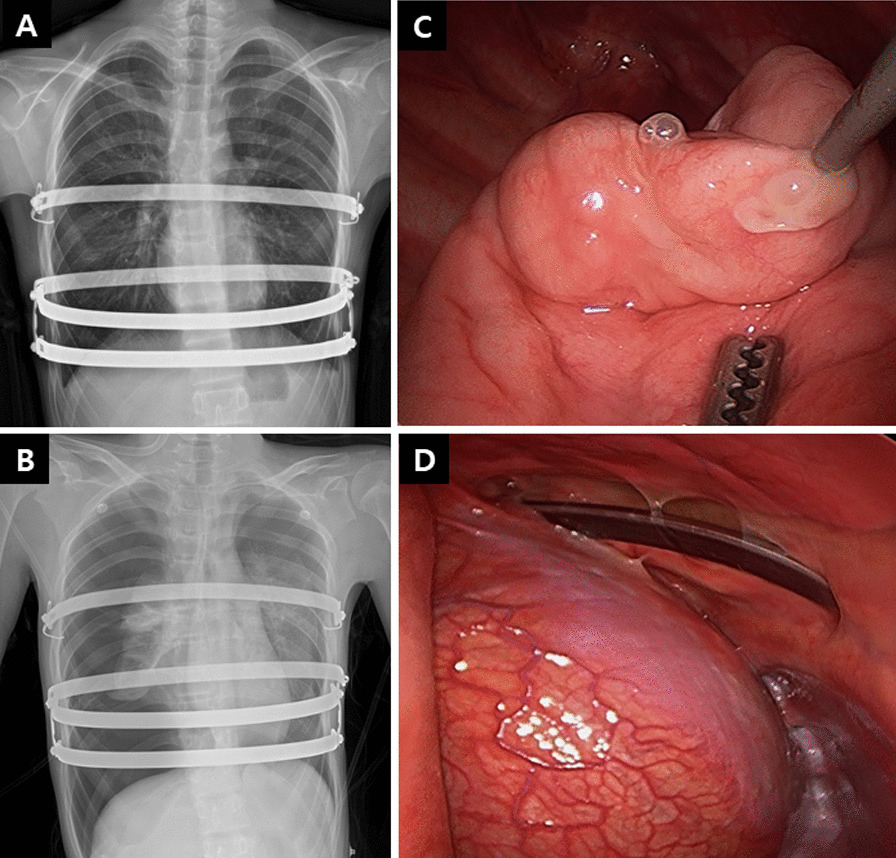
**A** A chest radiograph after Nuss procedure showing metal bars inserted for correction of pectus excavatum, with no surgical complications. **B** A chest radiograph during a visit to the emergency room demonstrating severe collapse of both lungs compressing the heart. **C**, **D** Intraoperative photos revealing several bullae in the upper lobe of the right lung and a mediastinal fenestration with an inserted metal bar

Buffalo chest, in which the left and right chest cavities are connected to each other through the mediastinal fenestration, can be of an iatrogenic or idiopathic etiology in humans. Schorlemmer et al. reported bilateral PSP that occurred during subclavian vein catheterization after median sternotomy [3]. In addition, bilateral PSP occurred after intrathoracic surgery including pneumonectomy, thymectomy, coronary artery bypass, bilateral lung or heart–lung transplantation, Nuss procedure, and esophagectomy [4]. In our case, bilateral tension pneumothoraxes occurred after the Nuss procedure, which was not related to any surgical complications. Although we do not explore the lungs searching for blebs during Nuss procedure in all patients who underwent pectus excavatum surgery, we always check the presence of blebs on chest CT before and after surgery and explain the relationship between pectus excavatum and primary pneumothorax to patients and their families.

Kılıçgün et al. [5] measured the Haller index, a ratio of the internal thoracic width and height, in 20 PSP patients and in 20 patients who underwent chest CT scans for other reasons. The Haller index of the PSP patient group was significantly higher than that of the other group, suggesting that more severe PE may be a predisposing factor in the development of PSP.

In summary, we encountered a life-threatening case of bilateral tension pneumothoraxes in the buffalo chest after the Nuss procedure for PE, which was not related to surgical complications. Together with the previous reports, our case further emphasizes that PE may be a predisposing factor of PSP, and PSP that occurs after the Nuss procedure, which causes a buffalo chest by inserting a metal bar into the substernal space, can be life-threatening.

## Data Availability

Not applicable.

## Abbreviations

PSP: Primary spontaneous pneumothorax
PE: Pectus excavatum
CT: Computed tomography
